# Isosilybin B: a potential novel therapeutic agent with hepatoprotective, anticancer and antifibrotic properties

**DOI:** 10.1007/s12672-025-03380-8

**Published:** 2025-08-08

**Authors:** Michal Selc, Kristina Jakic, Radka Macova, Andrea Babelova

**Affiliations:** 1 Centre for Advanced Material Application, Slovak Academy of Sciences, Bratislava, Slovakia; 2 Department of Nanobiology, Cancer Research Institute, Biomedical Research Center, Slovak Academy of Sciences, Bratislava, Slovakia; 3 Department of Genetics, Faculty of Natural Sciences, Comenius University Bratislava, Bratislava, Slovakia

**Keywords:** Hepatocellular carcinoma, Fibrosis, Isosilybin B, Silibinin, Silymarin

## Abstract

**Background:**

*Silybum marianum* (milk thistle) is a plant for centuries well known for its hepatoprotective effects. The extract from seeds, silymarin, and its major compound, silibinin, are well studied for their hepatoprotective and antifibrotic effects. The role of other minor compounds, such as isosilybin B, remains underexplored.

**Purpose:**

This study aimed to compare the cytotoxic and antifibrotic properties of IB with those of silibinin and silymarin in vitro. It focuses on evaluating the cytotoxic effect of these substances on tumor and non-tumor liver cells. Moreover, antifibrotic potential of the three substances was determined in healthy liver cells treated with TGF-β1.

**Results:**

Isosilybin B exhibits greater cytotoxicity toward liver cancer cells while being less toxic to non-tumor hepatocytes compared to silibinin. At non-toxic concentrations, isosilybin B induced cell cycle arrest at the G1 phase in two types of liver cancer cells. In contrast, it did not impact the cell cycle of non-tumor cells under the same experimental conditions. In the model of liver fibrosis in vitro induced by TGF-β1, isosilybin B reduced the mRNA expression of pro-fibrotic genes as well as ALT level in the culture medium more effectively than silibinin.

**Conclusion:**

Obtained results suggest that isosilybin B represents a promising anticancer agent for the treatment of liver cancer. Moreover, its anti-fibrotic properties emphasize its potential for treatment of many other liver diseases, which underline the strong potential of isosilybin B in future anticancer and antifibrotic therapeutic strategies.

**Supplementary Information:**

The online version contains supplementary material available at 10.1007/s12672-025-03380-8.

## Introduction

The medical use of *Silybum marianum*, commonly known as milk thistle, and its major compound– silibinin (SB), is well-documented for the treatment of various liver conditions including metabolic dysfunction-associated steatotic liver disease, alcoholic liver disease, viral hepatitis, cirrhosis, drug-induced liver injury, mushroom poisoning, and liver cancer [1, 2]. Liver diseases may result in hepatocellular carcinoma, a main type of liver tumor, with only limited treatment options. Thereby, liver cancer was the sixth most commonly diagnosed cancer globally and the third leading cause of cancer-related deaths in 2022 [3]. Within the intensive search for the novel treatment options to fight the liver carcinoma, SB has been considered a suitable candidate thanks to its antifibrotic, anti-inflammatory, antioxidative, or antiviral properties [1, 2]. Interestingly, a silibinin-based drug has already been approved for the treatment of liver intoxication following mushroom poisoning [4], and several others are in clinical testing phases [5–8]. Health benefits of SB are underlined by its use as a nutritional supplement to improve and support liver function. SB, a mixture of silybin A and B in a 1:1 ratio, typically accounts for approximately 40–60% of the total silymarin content. In addition to SB, silymarin (SM) contains several other flavonolignans and flavonoids, primarily silychristin (15–25%), silydianin (10%), isosilybin A (10%), 2,3-dehydrosilybin (5%), taxifolin (3%), isosilybin B (< 5%), and isosilychristin (3%) [9, 10]. All of these components possess antiviral, anti-inflammatory, and antioxidant properties. Among them, isosilybin B (IB) stands out because its cytotoxic effect in liver carcinoma cells occurs in concentrations eight-fold lower than other SM compounds [11]. IB has also been shown to induce apoptosis and inhibit growth of prostate cancer cells by causing G1 arrest, which was less pronounced in non-tumor cells, indicating selective action against cancer [12].

Based on already published data, isosilybin B could be, next to silibinin, also a suitable candidate for the treatment of liver diseases, but the data are incomplete, yet. Therefore, the purpose of this study was to compare the effects of IB, SB, and SM in non-tumor and tumor liver cells in vitro.

## Materials and methods

### Cell cultures

Mouse liver hepatoma cell line Hepa 1–6 (RRID: CVCL_0327, Cytion, Germany) was cultivated in Dulbecco’s Modified Eagle Medium/Nutrient Mixture F-12 (DMEM/F12) medium supplemented with 10% Fetal bovine serum (FBS), 1% penicillin-streptomycin. Human liver hepatocellular carcinoma cell line HepG2 (RRID: CVCL_0027, ATCC, USA) was cultivated in RPMI medium supplemented with 10% FBS and 1% penicillin-streptomycin. Mouse normal liver hepatocyte cell line AML12 (RRID: CVCL_0140, ATCC, USA) was cultivated in DMEM/F12 medium supplemented with 10% FBS, 1% penicillin-streptomycin, 1X Insulin-Transferrin-Selenium and 40 ng/mL dexamethasone. All chemicals used in the cell culture were from Gibco (Thermo Fisher Scientific, UK). Cell cultures were maintained in sterile humidified incubator (5% CO_2_, 37 °C).

### Reagents

Isosilybin B (#HY-N7045; MedChemExpress, USA), silibinin (#S0417; Sigma-Aldrich, USA) and silymarin (#05135001; Merck, Germany) were obtained from Lambda Life, Slovakia. All chemicals were dissolved in DMSO (#2438; Sigma-Aldrich, USA) to concentration 100 mg/mL and aliquots were stored at -20 °C. For experiments, stocks solution was 10X diluted in FBS and further diluted as needed. TGF-β1 (#240-B; R&D Systems, USA) was dissolved according to manufacturer´s recommendations.

### Cell viability

Cells were seeded in 96 well plate (1.5 × 10^4^ cells/well) and kept in medium with 10% FBS for 24 h at 37 °C. Then, the medium was replaced with medium containing 2% FBS and IB/SB/SM (0–250 µg/mL; the final concentration of DMSO in the culture medium did not exceed 0.25%). After 24 h incubation at 37 °C, medium was discarded, cells were washed with PBS and 150 µL media with PBS (in ratio 2:1) with 0.33 mg/mL MTT (#M5655, Sigma-Aldrich, USA) were added to cells and incubated for 3 h at 37 °C. Finally, medium was removed, formazan crystals in wells were dissolved in 100 µL DMSO and absorbance was measured at 540 nm using xMark™ Microplate Absorbance Spectrophotometer (Bio-Rad, USA).

### Cell cycle analysis

Cells were seeded in 6 well plate (2 × 10^5^ cells/well) and cultured for next 24 h at 37 °C, in medium with 10% FBS. After that, the medium was replaced with serum-free medium and cells were incubated for 16 more hours at 37 °C. Then, the medium was replaced with medium containing 10% FBS and IB/SB/SM (31.3 µg/mL). The selected concentration (31.3 µg/mL) represented the highest non-cytotoxic dose for all tested compounds, as determined in MTT viability assays. Following 24 h incubation at 37 °C, cells were washed with ice-cold PBS, trypsinized and centrifuged for 5 min at 250×g. The pellet was treated with Triton-X100 and 0.5 mg/mL RNase A (Sigma-Aldrich, USA) in the dark for 20 min at 37 °C, and then 6 µg/mL propidium iodide (Sigma-Aldrich, USA) was added. The cells were analyzed using the BD FACSCanto™ II flow cytometry system (BD Biosciences, USA) and results were evaluated using FCS Express software (De Novo Software).

### qRT-PCR

AML 12 cells were seeded in 6-well plate (2 × 10^5^ cells/well). After 24 h incubation at 37 °C, the cells were incubated with IB/SB/SM (7.8–31.3 µg/mL) for additional 24 h at 37 °C, in medium with 2% FBS. Total RNA was isolated using TRI-reagent (Sigma-Aldrich, USA) and treated with DNase I, RNase-free (Thermo Fisher Scientific, UK). cDNA was prepared from 1 µg of total RNA utilizing RevertAid First Strand cDNA Synthesis Kit (Thermo Fisher Scientific, UK). Semi-quantitative real-time PCR was conducted using FastStart Universal SYBRGreen Master (Rox) (Roche, Switzerland) and Aria MX PCR cycler (Agilent Technologies, USA). The primer sequences used in this study are detailed in Table 1. Primer specificity was confirmed by melting curve analysis and agarose gel electrophoresis. Relative expression levels of individual genes were normalized to *Actb* and calculated using the 2^−ΔΔCT^ method, presented as a ratio of treated to control samples.

**Table 1:** Primer list

*Fn1*	forward	5’-ATGCACCGATTGTCAACAGA-3’
	reverse	5’-TGCCGCAACTACTGTGATTC-3’
*Acta2*	forward	5’-CACCATGTACCCAGGCATTG-3’
	reverse	5’-GGCCCAGCTTCGTCGTATTC-3’
*Col1a1*	forward	5’-GTCCCAACCCCCAAAGAC-3’
	reverse	5’-CATCTTCTGAGTTTGGTGATACGT-3’
*Actb*	forward	5’-CAAGTACTCTGTGTGGATCGG-3’
	reverse	5’-TGCTGATCCACATCTGCTGG-3’

### Western blot analysis

Proteins from the media were precipitated using trichloroacetic acid and acetone, resuspended in SDS sample buffer, and boiled at 100 °C for 5 min. 40 µl of samples were loaded onto a 10% polyacrylamide gel and separated by SDS-PAGE. Subsequently, proteins were transferred onto a nitrocellulose membrane (Amersham, UK). The membrane was blocked with 1X ROTI (Carl Roth, Germany) and incubated overnight with a primary antibody against fibronectin (F3648; Sigma-Aldrich, USA) at 4 °C. After washing with TBS-T, the membrane was incubated with a secondary Goat anti-Rabbit IgG Alexa Fluor™ 680 antibody (A21109, Invitrogen, USA). Signal was visualized using the Odyssey imaging system (LI-COR Biosciences, USA), and protein relative expression change were evaluated using the Image Studio software (LI-COR Biosciences, USA).

### DPPH radical scavenging capacity determination

The absorbance change of 1,1-diphenyl-2-picrylhydrazyl radical (DPPH; Sigma-Aldrich, USA) was measured in a reaction mixture containing silymarin, silibinin, or isosilybin B, starting with an initial concentration of 500 µg/mL and using subsequent two-fold dilutions. A volume of 10 µL from each test compound solution diluted in methanol (Slavus, Slovakia) was mixed with 190 µL of DPPH solution (20 mg/L in methanol) in a 96-well microplate. The reaction mixtures were incubated in the dark at room temperature for 30 min, and the absorbance was measured at 517 nm using xMark™ Microplate Absorbance Spectrophotometer (Bio-rad, USA).

### ELISA

ALT level was measured in AML12 cell medium supernatant using Mouse ALT SimpleStep ELISA^®^ kit (ab282882, Abcam, USA) according to manufacturer´s recommendations. Samples of medium after treatment were collected, centrifuged and supernatant was stored at − 80 °C.

### Statistical analysis

The data represent means ± SD from three independent experiments. The One-Way ANOVA followed by Dunnett’s multiple comparisons test, or the two-way ANOVA followed by Tukey’s posthoc test was used to determine the statistical significance. Differences were considered significant at a *p < 0.05, **p < 0.01 or ***p < 0.001.

## Results

### Isosilybin B has better anticancer properties than Silibinin

**Fig. 1:**
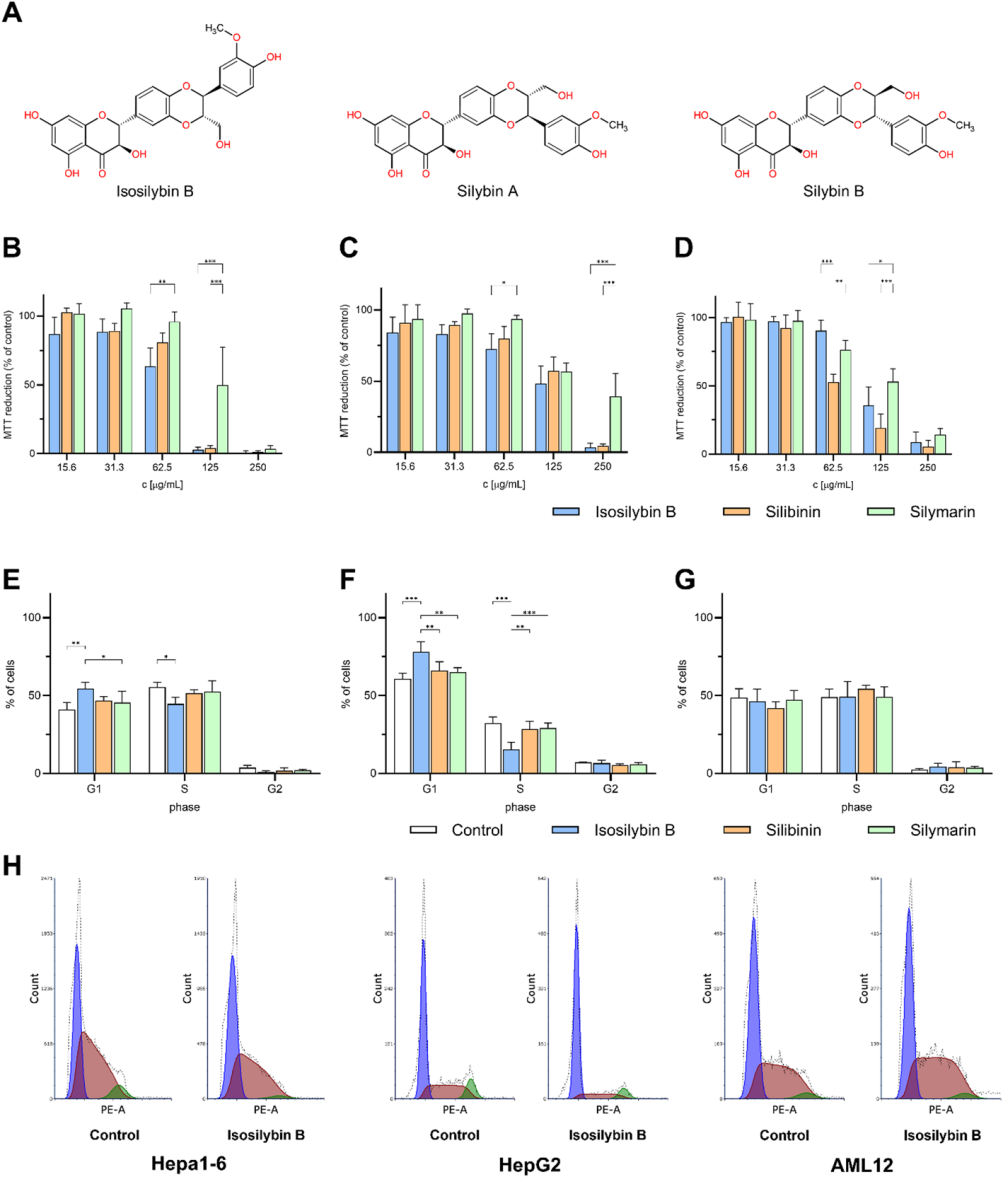
Effect of IB/SB/SM on cytotoxicity and cell cycle arrest of liver cells. *A*: Chemical structure of IB and SB (silybin A and silybin B in ratio 1:1). Cytotoxicity in Hepa1-6 (*B*), HepG2 (*C*) or AML12 (*D*) liver cells after 24 h treatment with IB/SB/SM. Changes in the cell cycle in Hepa1-6 (*E*), HepG2 (*F*), or AML12 (*G*) liver cells after 24 h treatment with 31.3 µg/mL of IB/SB/SM. *H*: Cell distribution of diploid cells in the phases of the cell cycle shown for IB (31.3 µg/mL). The data are given as means ± SD. *p < 0.05, **p < 0.01, ***p < 0.001

To determine the cytotoxic effect of SM and its components SB and IB (Fig. 1A) in liver cancer, mouse tumor Hepa 1–6 and human tumor HepG2 cells were treated with these substances for 24 h. IB reduced cell viability more strongly than SM in both Hepa 1–6 (Fig. 1B) and HepG2 (Fig. 1C) cells, with the most significant changes observed at 62.5 µg/mL. Higher concentrations of both IB and SB lead to complete cell death, which however was not true for SM (Fig. 1B&C). Interestingly, IB was much less toxic to non-tumor AML12 cells than SB (Fig. 1D). To quantify cytotoxicity, IC₅₀ values were calculated from three independent experiments using nonlinear regression (four-parameter logistic model). In non-tumor AML12 cells, IC₅₀ values were 124 ± 12 µg/mL (SM), 65 ± 3 µg/mL (SB) and 108 ± 9 µg/mL (IB). In Hepa1-6 liver tumor cells, IC₅₀ values were 123 ± 16 µg/mL (SM), 78 ± 2 µg/mL (SB) and 70 ± 3 µg/mL (IB). In HepG2 human liver cancer cells, IC₅₀ values were 174 ± 43 µg/mL (SM), 133 ± 9 µg/mL (SB) and 121 ± 15 µg/mL (IB). These results indicate that IB exhibit stronger cytotoxicity toward tumor liver cells than SM or SB, while IB demonstrates lower cytotoxicity toward non-tumor AML12 hepatocytes compared to SB, which underlined its potential as an anti-tumor drug. Changes in the cell cycle were observed after 24 h of exposure to IB/SB/SM at a non-toxic concentration of 31.3 µg/mL. In both Hepa1-6 and HepG2 cells IB induced cell cycle arrest in the G1 phase, while concurrently decreasing the S phase (Fig. 1E&F). These effects were not observed after SM and SB treatment (Fig. 1E&F). As expected, no alterations in the cell cycle due to IB were detected in AML12 cells (Fig. 1G&H). Therefore, the impact of isosilybin B on cell cycle arrest in liver cells appears to be tumor cell-specific.

### Isosilybin B reduces expression of pro-fibrotic genes after TGF-β1 stimulation

**Fig. 2:**
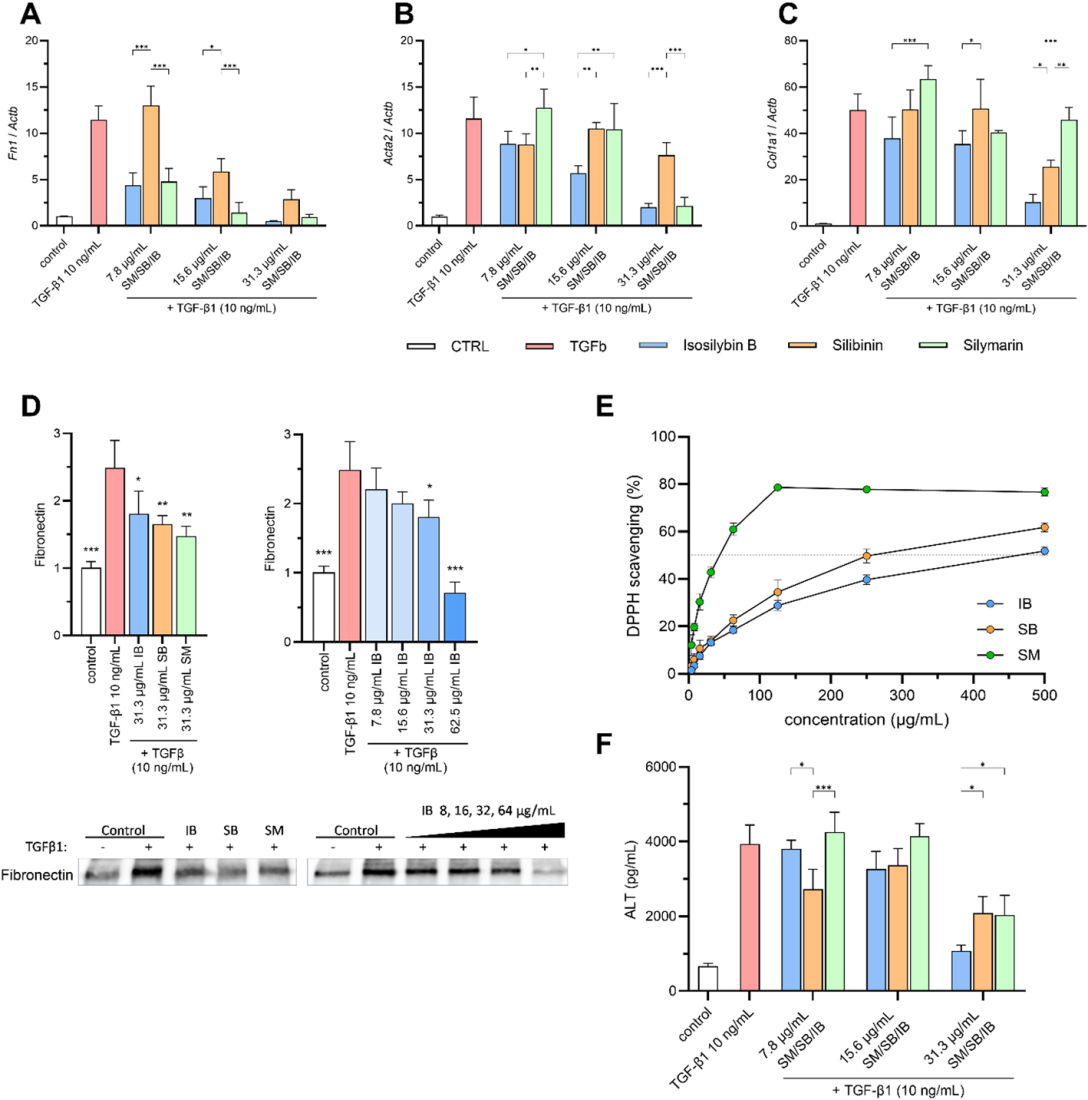
Effect of IB/SB/SM on pro-fibrotic gene expression, antioxidant activity, and ALT concentration in AML12 cells. (A-C) Expression of pro-fibrotic genes fibronectin (*Fn1*), smooth muscle actin (*Acta2*) and collagen I (*Col1a1*) after 24 h treatment with TGF-β1 (10 ng/mL) and IB/SB/SM (7.8–31.3 µg/mL) in AML12 cells. (D) Western blot analysis of fibronectin expression in AML12 cells stimulated for 24 h with TGF-β1 (10 ng/mL) and IB/SB/SM at concentration 31.3 µg/mL. Representative western blot bands are shown below the graphs. (E) DPPH antioxidant activity assay. The ability of IB/SB/SM to scavenge DPPH radicals was evaluated at various concentrations (0–500 µg/mL). (F) Determination of alanine aminotransferase (ALT) concentration in the supernatants of AML 12 cells stimulated with TGF-β1 (10 ng/mL) in the presence of the tested compounds (IB, SB, SM) at different concentrations. All results are expressed as mean ± SD, *p < 0.05, **p < 0.01, ***p < 0,001 (for western blot: vs. TGF-β1 control)

Both SM and SB possess significant antifibrotic properties in vitro and in vivo [13, 14]. The antifibrotic effects of IB, however, have not been studied yet. Therefore, our next aim was to examine the antifibrotic activity of IB and compare it with that of SM and SB. We used an in vitro model of AML12 cells incubated with recombinant TGF-β1 that led to increased expression of profibrotic genes. The most effective reduction of TGF-β1-induced fibronectin (*Fn1*) mRNA expression after 24 h was observed with SM and IB. SB showed the weakest decrease in *Fn1* mRNA (Fig. 2A). Interestingly, IB-induced reduction of smooth muscle actin (*Acta2*) and collagen I (*Col1a1*) was the strongest of the three tested substances (Fig. 2B, C). Nevertheless, prominent IB-mediated decrease in mRNA expression of profibrotic genes was associated with weaker reduction of fibronectin protein level in the cell culture medium after 24 h compared to SM and SB (Fig. 2D left, Supplementary material 1 - left). However, this reduction was IB-dose-dependent (Fig. 2D right, Supplementary material 1 - right).

### Isosilybin B, despite lower antioxidant activity, effectively decreases ALT levels in hepatocytes

To determine whether the three substances could attenuate the damaging impact of oxidative stress, the antioxidant activity using the DPPH assay has been evaluated. SM showed the highest antioxidant activity (IC_50_ = 40 µg/mL), followed by SB (IC_50_ = 250 µg/mL), and IB (IC_50_ = 500 µg/mL) (Fig. 2E). Despite its lower antioxidant capacity compared to SM and SB, IB has the most significant reduction in ALT levels of all the compounds tested, suggesting that its hepatoprotective effect may be mediated by mechanisms beyond direct antioxidation. To evaluate the protective effects of IB on liver cells, the levels of alanine aminotransferase (ALT) in the culture medium from AML12 cells treated with TGF-β1 for 24 h were monitored. Incubation of cells with IB led to a dose-dependent reduction in ALT levels. Although incubation with SM and SB also led to lower ALT levels following TGF-β1 stimulation, IB exhibited the highest reduction in ALT levels at a concentration 31.3 µg/mL compared to SM and SB (Fig. 2F) proving its strong hepatoprotective potential.

## Discussion

The cytotoxic effects of IB on liver tumor cells were stronger than those of SM and SB while exhibiting lower toxicity towards non-tumor hepatocytes, suggesting its remarkable potential as an anti-tumor agent. These results are consistent with previously published study showing isosilybin B-induced toxicity in tumor prostate cells, but not in non-tumor prostate cells together with stronger reduction of prostate-specific antigen level compared to silybins [12, 15], which has been associated with increased apoptosis [16]. The cell cycle analysis confirmed that a non-toxic concentration of IB induces G1 phase arrest in liver tumor cells. This effect was not observed after SM or SB treatment, suggesting a potentially unique tumor-selective activity of IB. This is in line with already published IB-induced HepG2 cell cycle arrest that occurred with half the concentration of SB in one-third of the time necessary for SB-mediated cell cycle arrest [17]. In addition, this IB activity is tumor-specific, as there was no cell cycle arrest in normal hepatocytes observed due to IB. This is confirmed by the previous results reporting cell cycle arrest in the G1 phase in prostate cancer cells by IB without affecting non-tumor cells [12].

Regarding antifibrotic properties, IB demonstrated activity comparable to, and in some cases greater than, that of SM and SB in reducing the expression of profibrotic genes such as *Fn1*, *Acta2*, and *Col1a1.* However, IB exhibited a weaker reduction in fibronectin protein levels, suggesting a possible post-transcriptional regulatory mechanism. The development and progression of liver fibrosis are commonly associated with oxidative stress [18], we assessed the antioxidant activity of all three substances using the DPPH assay. Our results align with the literature, although previous findings are somewhat inconsistent. IB exhibited greater antioxidant activity than SB in one study [19], while it was less effective in another [20]. Nonetheless, the strongest DPPH activity consistently observed for SM, likely due to the presence of taxifolin, a potent antioxidant [19, 20]. Interestingly, although IB demonstrated the weakest antioxidant potential in the DPPH assay, its ability to reduce ALT levels was comparable to or even greater than that of SB or SM in some conditions. As shown by Zhang et al. (2015), phenolic compounds with strong DPPH activity do not necessarily exhibit high anti-lipid-peroxidation capacity, and vice versa. For instance, resveratrol showed poor DPPH scavenging but effectively prevented lipid peroxidation [21], and was also shown to significantly lower ALT *levels* in vivo [22]. Moreover, anti-lipid peroxidation strongly correlates with reduced serum ALT and AST, suggesting that DPPH-based assays may not fully capture the biological antioxidant capacity of a compound, especially in lipid-rich organs such as the liver [23].

In our model, TGF-β1-induced injury was not primarily oxidative, but profibrotic. Thus, the observed reduction in ALT may stem from antifibrotic effects of IB, as it significantly downregulated TGF-β1-responsive genes. At the highest non-toxic concentration (31.3 µg/mL), IB most effectively reduced ALT levels in AML12 cells while simultaneously decreasing Fn1, Acta2, and Col1a1 expression. This suggests that its ALT-lowering effect may result from antifibrotic rather than antioxidant action. In addition, this effect may be linked to the ability of IB to lower triglyceride accumulation, which is closely associated to liver cell damage and ALT elevation. Excess accumulation of triglycerides leads to steatosis, an early indicator of metabolic dysfunction-associated steatotic liver disease [24]. IB was previously shown to reduce triglycerides in HepG2 cells exposed to free fatty acids, while also modulating lipid metabolism-related gene expression [25]. The combination of antifibrotic, hepatoprotective, and triglyceride-lowering effects makes IB a promising candidate for the treatment of early stages of liver disease, similarly as SM and SB, but potentially with additional advantages. The ability of IB to selectively induce the G1 cell cycle arrest in the liver cancer cells but not in non-tumor hepatocytes, which was not observed with SM and SB, suggests its potential role in anticancer therapy targeting hepatocellular carcinoma. However, future studies should investigate these mechanisms in more depth and shift the focus towards in vivo models.

Although IB shares the same atomic composition as SB, it differs in stereochemistry, existing as the 2R, 3R, 7’S, 8’S diastereomer. For comparison, silybin A has the 2R, 3R, 7′R, 8′R configuration, silybin B is 2R, 3R, 7′S, 8′S, and isosilybin A is 2R, 3R, 7′R, 8′R [26]. This structural variation may underlie its distinct biological behavior. Stereochemistry is known to influence both pharmacokinetics and pharmacodynamics, affecting molecular interactions, cellular uptake, and metabolic stability [27]. In a pharmacokinetic study conducted in healthy volunteers, Zhu et al. (2013) observed stereoselective differences in the clearance of flavonolignans: isosilybin B exhibited significantly lower clearance than isosilybin A, and silybin B was cleared more rapidly than silybin A [28]. These findings support the hypothesis that the unique stereochemistry of isosilybin B contributes to its favorable pharmacological profile, distinguishing it from other silymarin constituents. Therefore, exploring the therapeutic relevance of lesser-studied flavonolignans and related compounds may uncover new opportunities in the treatment of liver diseases.

## Conclusion

Isosilybin B is found in milk thistle at approximately one-tenth the quantity of silibinin [9, 10]. However, our results showed its better anticancer and similar antifibrotic properties in liver cells in vitro compared to SB. Especially, the weak cytotoxic effect of IB on non-tumor hepatocytes compared to SB and SM suggests desired selective anti-tumor activity. Therefore, isosilybin B appears to be a promising candidate for research in the biomedical field and future anticancer or antifibrotic strategies.

## Supplementary Information

Below is the link to the electronic supplementary material.

Supplementary Material 1

## Data Availability

The data used to support the findings of this study are available from the corresponding author upon request.
