# Supplementary material for: Isosilybin B: a potential novel therapeutic agent with hepatoprotective, anticancer and antifibrotic properties
Source: Discov Oncol. 2025 Aug 8;16:1502. doi: 10.1007/s12672-025-03380-8 (PMC12334778; doi:10.1007/s12672-025-03380-8)
Supplement: Supplementary file 1 — Supplementary Material 1 [file 12672_2025_3380_MOESM1_ESM.docx]

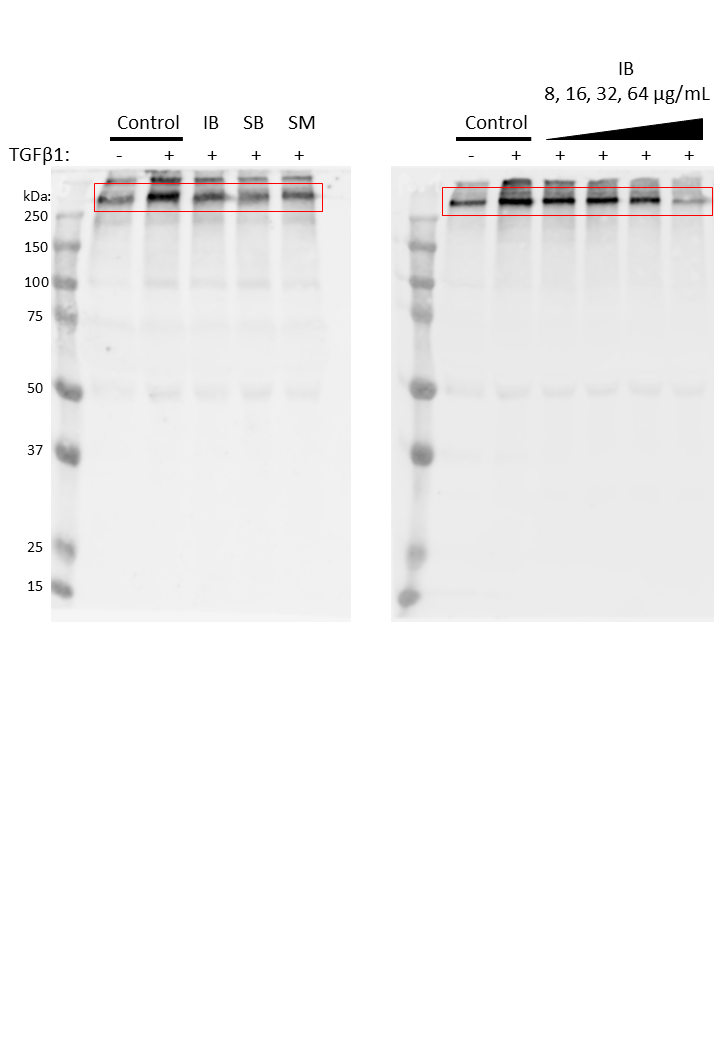


**Supplementary material 1.** Uncropped Western blot images corresponding to Fig. 2D. Full-length images of fibronectin blots from AML12 cells treated with TGF-β1 (10 ng/mL) and either isosilybin B (IB), silibinin (SB), or silymarin (SM) at concentration 31.3 µg/mL. Red boxes indicate the regions shown in the main figure. These images were obtained using the Odyssey imaging system.
